# Monitoring Fish Bacterial Pathogens of Wild Fish Species From the South China Sea by Applying Next‐Generation Sequencing on Gill Tissue

**DOI:** 10.1111/jfd.14050

**Published:** 2024-11-22

**Authors:** Shlomi Zrihan, Peleg Itay, Yael Kroin, Nadav Davidovich, Natascha Wosnick, Dan Tchernov, Xiu Pei Koh, Stanley C. K. Lau, Danny Morick

**Affiliations:** ^1^ Morris Kahn Marine Research Station University of Haifa Sdot Yam Israel; ^2^ Department of Marine Biology, Leon H. Charney School of Marine Sciences University of Haifa Sdot Yam Israel; ^3^ Department of Blue Biotechnologies and Sustainable Mariculture, The Leon H. Charney School of Marine Sciences University of Haifa Sdot Yam Israel; ^4^ Israeli Veterinary Services Bet Dagan Israel; ^5^ Programa de Pós‐Graduação Em Zoologia Universidade Federal Do Paraná Curitiba Brazil; ^6^ Department of Ocean Science The Hong Kong University of Science and Technology Hong Kong SAR China

**Keywords:** 16S rRNA, gill microbiome, next generation sequencing, South China Sea, wild fish pathogens

## Abstract

The classic epidemiological triangle model of host—environment—pathogen is recently being reshaped into a tetrahedron, with the growing understanding of the importance of the microbiome in this array. The gills, being a gateway into the fish body, bearing an important role in fish homeostasis, host a complex microbiome that reflects the ambient water, while also showing resemblance to gut microbiome. Next‐generation sequencing (NGS) and improvements in data analysis tools enable researchers to gather and analyse a lot more data than ever before, take a closer, more detailed look at microbiota, and gain a much better understanding of the biological processes at work in these complex relations. Here, 16S rRNA amplicons of bacterial DNA extracted from the gills of 36 asymptomatic specimens of three wild fish species from the South China Sea (*Nemipterus japonicus*, *Alepes djebaba,* and *Saurida tumbil*) were sequenced using NGS. Data analyses revealed the presence of 20 potentially pathogenic species, including several zoonotic agents. Gill microbiota exhibited host species‐specificity, and expressed a significant difference between demersal and pelagic‐amphidromous fish. It is suggested that this method be more widely implemented, in order to gain more insight on ocean ecosystems’ health status, as well as fish stocks of commercial importance.

## 1 Introduction

In a global climate rapidly changing and deeply affected by human activity (Vogel et al. 2019), the oceans are not exempt (Laufkötter, Zscheischler, and Frölicher 2020). With one sixth of the global animal protein intake being sourced from fish, half of which is wildly caught at sea, the impact of many stress factors on an important source of food for billions of people worldwide is of great concern (FAO 2024). The influence of pollution, sea temperature rise, and ocean acidification are some of the major factors posing risk to fish populations (Broekhuizen et al. 2021; Cheng et al. 2020; Finn, Grattarola, and Pincheira‐Donoso 2023; Pistevos et al. 2015; Viršek et al. 2017). In order to assess the ability of these populations to cope with the stress, researchers look at external physical and chemical factors, such as previously mentioned, as well as the internal biological aspects of fishes themselves, and their relationships with microbiological organisms affecting them through various commensal relations (Llewellyn et al. 2014). Disease outbreaks are more pronounced and measurable in aquaculture than in wild fish populations (Chapman et al. 2021; Clavelle et al. 2019), however the importance of either preventing or limiting the extent of these outbreaks has extreme economic and social implications (FAO 2024). Furthermore, since mariculture has been shown not only to affect the surrounding waters and wild fish populations within it, but also to be affected by it in a two‐way process (Arechavala‐Lopez et al. 2013), the importance of gathering real time data cannot be overstated. Therefore, monitoring of wild fish populations’ health can prove to be a relatively low‐cost indicator—one of several—which can be implemented in order to reduce economic losses and risk to public health. The epidemiological triangle, describing the relationship between the environment, a host and a pathogen (King et al. 2019), forms the foundations of research looking into the clockworks of marine animal diseases (Andrade et al. 2017; Elarabany et al. 2017; Genin et al. 2020; Wang et al. 2018; Zarantoniello et al. 2021). In recent years, another layer has been added to this model—the microbiome. The close partnership between hosts and their symbiotic microbiota plays a significant role in host maintenance and wellbeing, contributing to metabolism, immune system maturation, and additional defences against pathogenic invaders (Apprill 2017; Aschenbrenner et al. 2016; Ramsey et al. 2016; Vorburger and Perlman 2018). Changes in microbial composition due to internal (Yildirimer and Brown 2018) and/or external stress factors (Halpern et al. 2008; Nguyen and Liou 2019; Pérez‐Ruzafa, Pérez‐Marcos, and Marcos 2018), may have a major effect on the wellbeing of the host (Llewellyn et al. 2014). Hence, fish microbiota trends may serve as a bioindicator for the host's health status. Research of fishes’ microbiota has been growing in the past decade, with studies varying in the organ of focus, ranging from the skin to internal organs (digestive system, kidneys, liver, spleen, etc.) (Egerton et al. 2018; Krotman et al. 2020; Liu et al. 2016; Meron et al. 2020; Ni et al. 2013; Sevellec et al. 2014; Tarnecki et al. 2019), and recently increasingly more—the gills (Itay et al. 2022; Merrifield and Rodiles 2015; Mohammed and Arias 2015). The gills, being a gateway to the body and blood system, serve several purposes in the wellbeing of a fish: from their primary role in gas and waste exchange to being an important mucosal immunity site (Evans, Piermarini, and Choe 2005; Salinas 2015). They have been found to be relatively well‐correlated to gut microbiota (Pratte et al. 2018), and thus a good representative of the internal physiological state of the fish, as well as a proxy to ambient water bacteria community composition, pathogens included (Kuang et al. 2020). There are two practical reasons to use gills as the target organ for such monitoring programs: (i) harvesting gill tissue is less time consuming relative to internal organs, which enables handling more samples in a given time frame; and (ii) a small clipping of the tissue may be sufficient for examination; hence fish do not necessarily have to be killed in order to be studied. In this study, we implemented 16S rRNA NGS to screen the gills of three wild fish species, analyse their gills’ microbial community composition and search for potential pathogens. This method follows in the footsteps of similar studies previously performed (Itay et al. 2022), further supporting the case for implementing it in more regions as a cost‐effective monitoring tool (Caporaso et al. 2011; Vayssier‐Taussat et al. 2013; Walters et al. 2015).

## 2 Materials and Methods

### 2.1 Fish Collection

**FIGURE 1:**
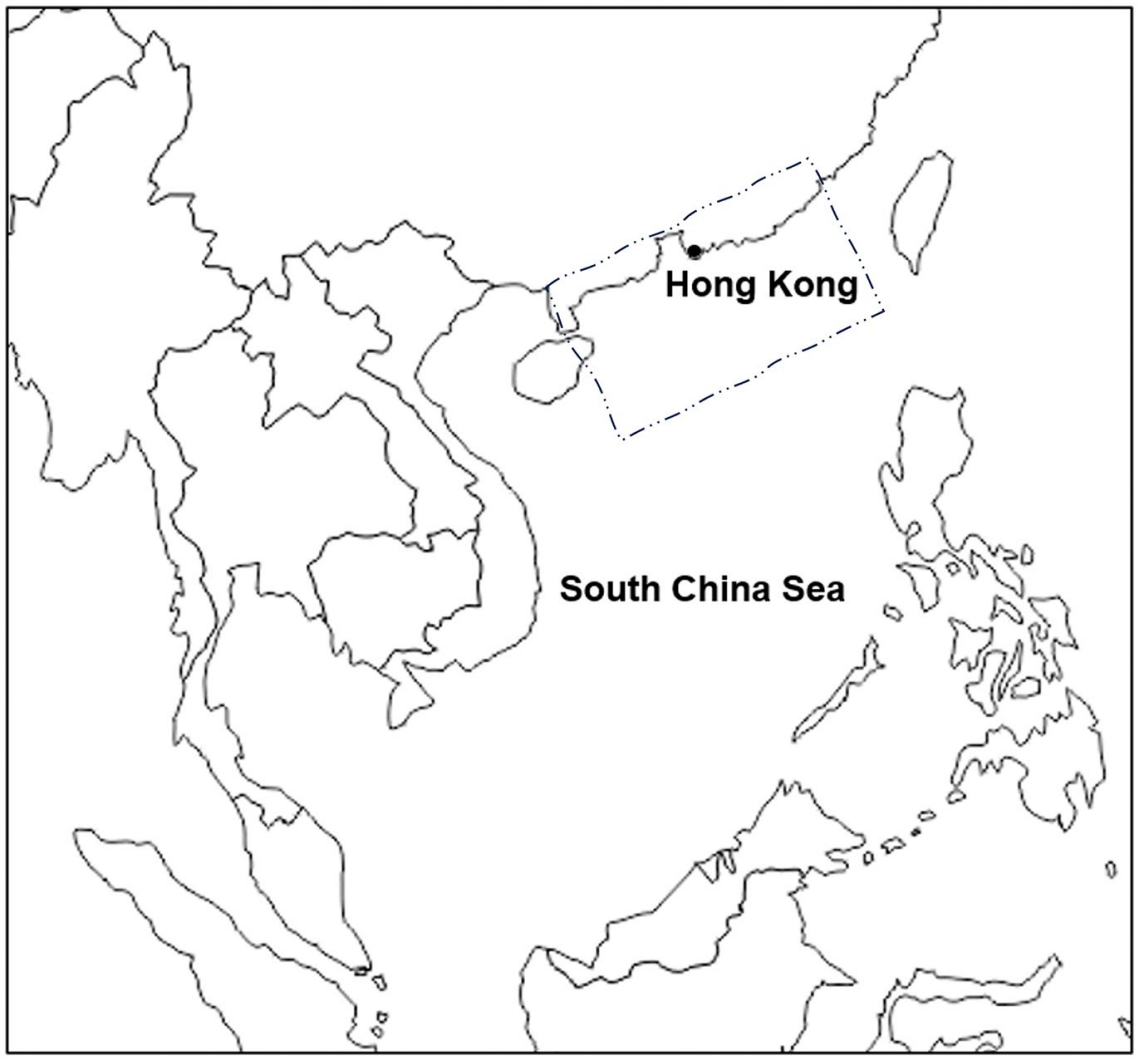
A map of the South China Sea, showing the area from which the fish samples originated. Map modified from: https://www.d‐maps.com/carte.php?num_car=79&lang=en.

Fish were caught by fishermen in Hong Kong, in the northern part of the South China Sea (Figure 1), and were bought to the laboratory in the Hong Kong University of Science and Technology from a stall in a local fish market. During that whole process—from the minute they were brought up by fishermen until they were sold, then later brought to the lab for dissection—the fish were kept on ice. All samples which were not immediately dissected were kept in a −20°C fridge until being processed. Japanese threadfin bream (*Nemipterus japonicus*; *n* = 11) and Shrimp scad (*Alepes djebaba*; *n* = 13) samples were fished during December 2020. Greater lizardfish samples (*Saurida tumbil*; *n* = 12) were collected during January 2021.

### 2.2 Tissue Sampling

Tissue sampling was done based on Yanong (2003) and according to the protocol in Supporting Information (Data [Link], [Link], [Link]). In short, frozen fish specimens were gradually thawed in small batches in order keep their inner organs in a state of partial thawing, which is ideal for removal. The fish were weighed and measured (data added in the Supporting Informations section Data [Link], [Link], [Link]), inspected externally and were then dissected aseptically. Gills samples (arc and filaments from a middle gill, one of each side) were placed in 2 mL‐tubes, which were kept frozen at −80°C until DNA was extracted from them.

### 2.3 DNA Extraction

DNA extractions were done using the GeneMATRIX Soil DNA Purification Kit (EURx, Gdańsk, Poland), following the manufacturer instructions for tissue lysates, with an extra two‐hour incubation at 55°C following suspension in the kit‐provided lysis buffer of the tissue sample. DNA quality was examined by NanoDrop spectrophotometry analysis and agarose gel‐electrophoresis. Extracted DNA samples were stored at −80°C and later couriered to Israel in a box of dry ice.

### 2.4 PCR Amplification and Amplicon Sequencing

Total DNA extracts were used as template for amplification of partial 16S rRNA gene sequences, at the V4 hypervariable region. Amplicon generation followed a two‐stage PCR amplification protocol (Naqib et al. 2018). Each of the first stage PCR reactions (a total of 50 μL in volume) included: 25 μL of GoTaq Green Master mix (Promega, Fitchburg, WI, USA), 2 μL of mixed forward‐reverse primers (each in a concentration of 1 nM), 2 μL of bovine serum albumin (BSA), 18 μL of ultra‐purified water (UPW) and 3 μL of 80 ng/μL template DNA. The primers contained 5′ common sequence tags (known as common sequence 1 and 2, CS1 and CS2) compatible with Access Array primers for Illumina sequencers (Fluidigm, South San Francisco, CA, USA) (Caporaso et al. 2012). Amplification primers used (linker sequences in **bold**): CS1_518F: 5′ –**ACACTGACGACATGGTTCTACA**CCAGCAGCCGCGGTAATACG—3′ (Nakasaki et al. 2009) and CS2_806Rc: 5′—**TACGGTAGCAGAGACTTGGTCT**GGACTACNVGGGTWTCT—3′ (Walters et al. 2015).

The PCR conditions were set as follows: 10 cycles of denaturation at 95°C for 15 s, annealing at 60°C for 15 s and elongation at 72°C for 30 s; followed by 10 cycles of denaturation at 95°C (15 s), annealing at 55°C (15 s) and elongation at 72°C (30 s); after which 10 more cycles at 95°C (15 s)/50°C (15 s)/72°C (30 s) were run; and then 5 final cycles with only a change of annealing temperature, performed at 62°C. The PCR concluded with 2 min of incubation at 72°C, before being lowered to 4°C for 1 h (or until samples were removed). Amplicons were sent to UIC Sequencing Core (Chicago, IL, USA), in which a second PCR amplification was performed in 10 μL reactions in 96‐well plates using MyTaq HS 2X mastermix (Bioline, Taunton, MA, USA). Each well received a separate primer pair with a unique 10‐base barcode, obtained from the Access Array Barcode Library for Illumina (Fluidigm, South San Francisco, CA; Item# 100‐4876). One μl of PCR product from the first stage amplification was used as template for the 2nd stage, without cleanup. Cycling was performed using the following conditions: 95°C for 5 min, then 8 cycles of 95°C for 30″, 60°C for 30″, and 72°C for 30″. Libraries were then pooled and sequenced using a 20% phiX spike‐in on an Illumina Miniseq sequencer employing a mid‐output flow cell (2 x 150 paired‐end reads). Final library preparation, pooling, and sequencing were performed at the genome research core (GRC) at the University of Illinois at Chicago (UIC).

### 2.5 Sequence Data Processing

Detailed information regarding the sequence data processing is provided in the Supporting Information File (Appendix S1). In brief, sequence data was analysed using the Dada2 pipeline (Callahan et al. 2016) using R package ‘dada2’ (version 1.14.1). Error rate estimation was carried out in order to sample nucleotides and reads for model building randomly across all samples. The dada2 algorithm was implemented for error correction and a count table containing the amplicon sequence variants and counts per sample was produced. For each amplicon sequence variant (ASV), taxonomy (up to the species level) was inferred by alignment to the Silva non‐redundant small subunit ribosomal RNA database (version 138).

### 2.6 Data Analysis

All data filtering parameter settings are detailed in the Supporting Information File (Appendix S1). In short, for data analysis and generation of figures, the online tool MicrobiomeAnalyst (https://www.microbiomeanalyst.ca/MicrobiomeAnalyst/home.xhtml) was used (Chong et al. 2020; Dhariwal et al. 2017). Taxonomy labels were assigned using the SILVA taxonomic framework (https://www.arb‐silva.de/documentation/silvataxonomy/). Initial analyses identified 251 unique bacterial species. The prevalence of these bacterial species was calculated per fish species, and ranged from a single occurrence in just one specimen of the whole pool of samples of the three fish species—to an appearance in nearly all samples. Furthermore, the accumulated number of reads per bacterial species was calculated. A certain correlation between increased occurrences and increased accumulated number of reads was observed, as expected. Hence, a cutoff of 100 total reads was used to frame the species of interest. This cutoff was based on the assumption that a total number of reads beneath that threshold can be considered relatively ‘safe’, in terms that a fish infected by a pathogenic bacterium with such a low number of reads is probably not in risk of developing a disease, as explained in detail in Itay et al. (2022). All 73 bacterial species with at least 100 total reads were then searched for pathogenic potential by adding the words ‘infection’, ‘disease’, ‘marine’, ‘fish’, and ‘human’ to their names in Google Scholar. According to the results of the search, each species was rated for its pathogenic potential from ‘No’, through ‘Unknown’, ‘Rarely’, ‘Opportunistic’, ‘Pathobiont’, ‘PP’ (meaning Potentially Pathogenic) to ‘Yes’, as described in Itay et al. (2022).

### 2.7 Phylogenetic Trees

The parameters used for creating trees are detailed in the Supporting Information file (Appendix S1). Briefly, sequences identified as belonging to the several genera chosen for deeper enquiry were uploaded to Silva (https://www.arb‐silva.de/aligner/) for preparing phylogenetic files (Oliver et al. 2017; Quast et al. 2013; Yilmaz et al. 2014). The ACT (Alignment, Classification and Tree Service) tool was used (SINA v1.2.11) (Pruesse, Peplies, and Glöckner 2012). Output TREE format files were extracted for visualisation with the FigTree v1.4.4 software (http://tree.bio.ed.ac.uk/software/figtree/).

## 3 Results

**FIGURE 2:**
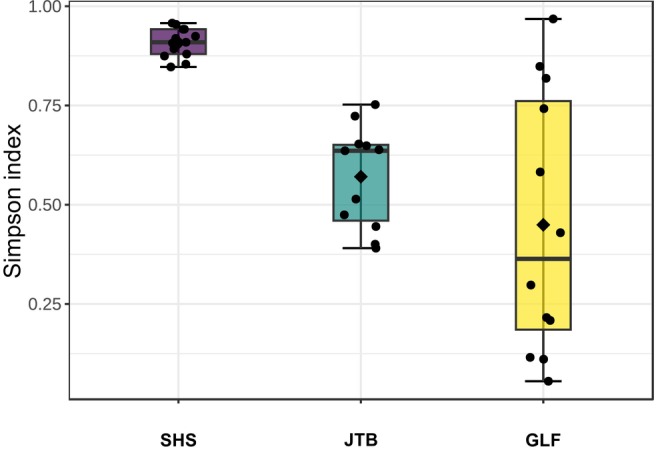
Community structure (aka: α‐diversity) profiling of fish gills samples. The Simpson diversity index boxplot shows data clustered by fish species. Kruskal‐Wallis statistic: 19.745, *p* < 0.5.158e‐05.

All of the 36 fish collected appeared healthy both externally and internally upon inspection and necropsy. The community structure of the gill samples differed between fish species (Figure 2). Shrimp scad (*Alepes djebaba*; hereafter ‘SHS’) samples exhibited a higher and richer composition (Simpson index average: 0.91), while also expressing the lowest variance between samples. Greater lizardfish (*Saurida tumbil*; hereafter ‘GLF’) samples exhibited high variance and low average, less rich compositions (averaging at 0.45 on the Simpson index). Japanese threadfin bream (*Nemipterus japonicus*; hereafter ‘JTB’) expressed a medium level variance and Simpson index (average at 0.57).

**FIGURE 3:**
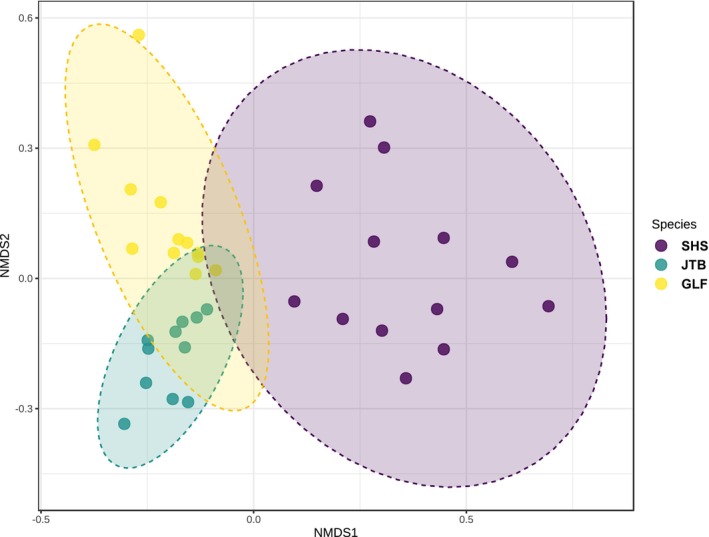
Gills’ microbiome resemblance between the sampled fish species. Jaccard index: *F*: 23.645, *R* ^2^: 0.5889, *p* < 0.001, NMDS stress = 0.13311.

A comparison of compositions (Figure 3) shows a clustering of microbiomes amongst species, with SHS displaying a community structure least similar to the other two, while JTB shares a large part of its microbiome with GLF.

**FIGURE 4:**
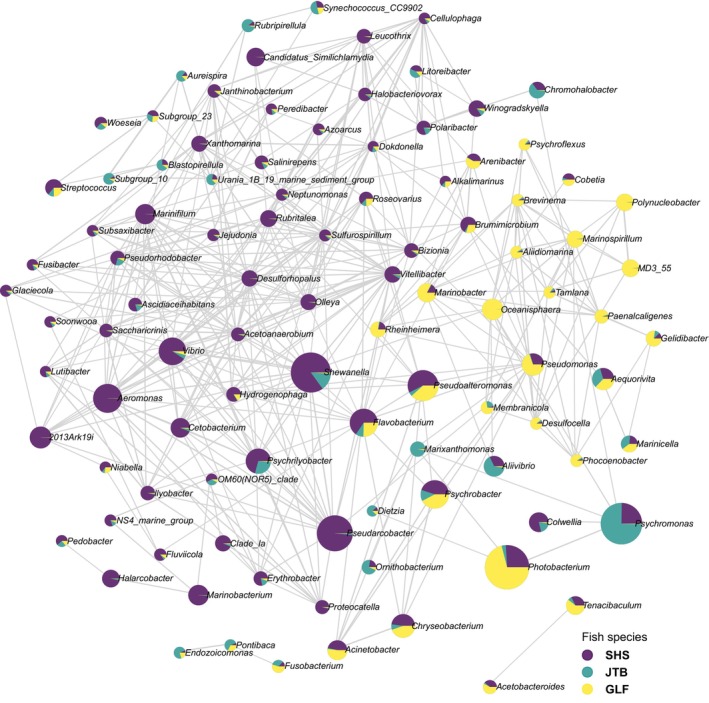
A network of interactions between different genera, with reference to three wild fish species from the South China Sea. Network calculation used Spearman's rank correlation coefficient and the threshold settings placed at: Correlation > 0.5; *p* < 0.05. Size of circles represents abundance and the colouring associates bacteria with host in regards to mean abundance.

An interaction network (Figure 4), expressing the strength of ties between bacteria genera to each other and their tendency to be hosted by the different fish species, reveals a complex structure with no distinct cohort. Nonetheless, some genera found in substantial numbers were more strongly associated with specific fish species rather than others: *Pseudarcobacter*, *Shewanella*, *Aeromonas*, *Vibrio*, *Cetobacterium*, *Flavobacterium* and *Psychrilyobacter* were predominately found in SHS gills; *Photobacterium*, *Tenacibaculum* and *Oceanisphaera* were common in GLF; while *Psychromonas* and *Aliivibrio* had shown up more in JTB samples. Most of the ties between bacteria genera were positive correlations.

**FIGURE 5:**
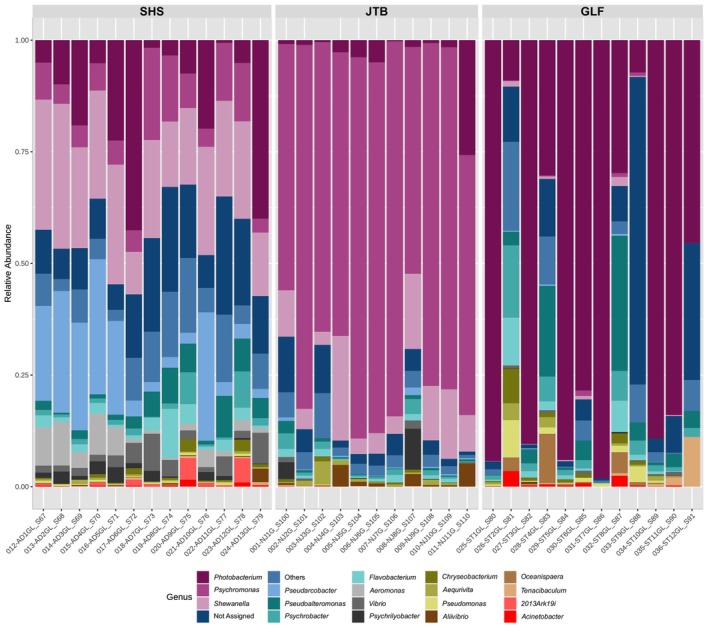
Relative abundance of bacterial species within each of the fish species’ gills. Small taxa with counts < 3000 were merged together (‘Others’).

The relative abundance analysis (Figure 5) shows that two of the most dominant genera, *Photobacterium* and *Psychromonas* were negatively correlated (−0.8232, according to the correlation network data). In GLF, *Photobacterium* was on average 64% of the gill's microbiome (of which 95% of the reads came from *P. leiognathi*, a non‐pathogenic bacterium), while *Psychromonas* made up less than 0.5%. In JTB, on the other hand, *Psychromonas* dominated 70% of the microbiome, while *Photobacterium* was only 4%. In JTB, other important members of the gill's microbiome included *Shewanella* (9.7% on average) and *Aliivibrio* (1.5%). Less than 5% of the JTB reads could not be assigned (NA; i.e., not identified to genus). In GLF, apart from the aforementioned *Photobacterium*, the genera *Pseudoalteromonas* (5.9%), *Psychrobacter*, *Flavobacterium*, and *Oceanisphaera* (4.5%, 3.4% and 2%, respectively) were noteworthy microbiome constituents. In this fish species on average 12.4% of the reads were NA. In SHS, the gills microbiome expressed a much more balanced picture, in the meaning that no single genus was so dominant. Nonetheless, *Shewanella* was the leading element, taking up on average as much as 21.5% of its gill's microbiome. Other important genera included *Photobacterium* (14.3%), *Pseudarcobacter* (13.2%), *Psychromonas* (8.5%), *Pseudoalteromonas* (3.92%), *Aeromonas* (3.89%), *Vibrio* (3.11%), *Flavobacterium* (2.65%), and *Psychrobacter* (1.92%). SHS had the largest percentage of NA (14.25%).

**FIGURE 6:**
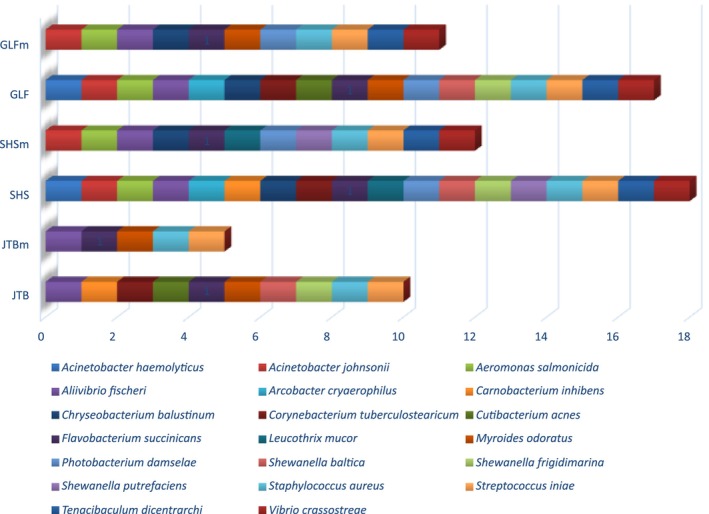
Number of potentially pathogenic bacterial species that were found in the gills of three fish species. A lower‐case ‘m’ to the right of a fish species’ abbreviation marks pathogens with a potential to affect marine animals. Hence, each fish species is represented by two rows ‐the upper one shows marine pathogens, and the lower one shows pathogens with human relevance.

The NGS data analyses provided a total output of 5655 unique bacterial amplicon sequence variants (ASVs) of which only one (ASV0005), identified as a *Pseudoalteromonas sp*., appeared in all fish samples. Of the whole list of ASVs, 251 were identified to the taxonomic level of (unique bacterial) species. Of these, 73 were found with at least 100 total reads and screened in the literature for their pathogenic potential (see methods for details). A list of 20 species bearing some pathogenic potential (to humans and/or marine organisms) was assembled (Table 1). These bacteria belong to 5 different classes: Gammaprteobacteria (*n* = 10); Bacteroidia (*n* = 4); Bacilli (*n* = 3); Acinetobacteria (*n* = 2) and Campylobacteria (*n* = 1). The list consists of 13 species recognised as carrying different degrees of pathogenicity to marine organisms, of which six are also known to bear some pathogenic potential to humans. Overall, there were 13 bacterial species with certain pathogenicity to humans. In samples belonging to SHS 18 out of the 20 (18/20) potentially pathogenic (PP) bacterial species appeared and 12 out of 13 bacterial species pathogenic to marine organisms. In GLF 16 PP species (11/13 marine pathogens) were found and in JTB samples 10 PP species (5/13) showed up. These results are visualised in Figure 6.

**TABLE 1:** List of potentially pathogenic bacteria to marine animals and humans.

Bacteria	JTB (%)	SHS (%)	GLF (%)	Marine pathogenicity	Marine pathology	Clinical relevance	Human pathology
*Shewanella frigidimarina*	100.0	100.0	66.7	Unknown		Yes	Food spoilage Wright et al. (2019)
*Shewanella baltica*	81.8	100.0	58.3	Unknown		Yes	Food spoilage Zhu et al. (2015)
*Aliivibrio fischeri*	100.0	76.9	8.3	Pathobiont	Liver haemorrhages, internal bleeding López et al. (2017)	Unknown	
*Streptococcus iniae*	72.7	53.8	41.7	Yes	Streptococcosis Chou et al. (2014)	Opportunistic	Meningitis, endocarditis, septic arthritis, osteomyelitis, toxic shock Soh et al. (2020)
*Vibrio crassostreae*	0.0	100.0	41.7	Opportunistic	Pacific oyster mortality syndrome Piel et al. (2020)	Unknown	
*Flavobacterium succinicans*	27.3	69.2	41.7	Opportunistic	Bacterial gill disease Good et al. (2015)	Unknown	
*Photobacterium damselae*	0.0	46.2	66.7	Yes	Haemorrhages, ulcerative lesions, septicemia Pham et al. (2020), Rivas, Lemos, and Osorio (2013), Terceti, Ogut, and Osorio (2016)	Opportunistic	Necrotizing fasciitis Matanza and Osorio (2020)
*Aeromonas salmonicida*	0.0	100.0	8.3	Yes	Skin ulcers, haemorrhages Menanteau‐ledouble et al. (2016)	Rarely	Gastroenteritis Vincent et al. (2019)
*Shewanella putrefaciens*	0.0	84.6	0.0	Pathobiont	Shewanellosis Esteve, Merchán, and Alcaide (2017), Paździor (2016)	Rarely	Intra‐abdominal, skin and soft tissue infections Yu et al. (2022)
*Tenacibaculum dicentrarchi*	0.0	7.7	75.0	Yes	Severe skin lesions Saldarriaga‐Córdoba, Irgang, and Avendaño‐Herrera (2021)	Unknown	
*Acinetobacter haemolyticus*	0.0	69.2	8.3	Unknown		Yes	Endocarditis, bloody diarrhoea Figueiredo et al. (2012)
*Chryseobacterium balustinum*	0.0	46.2	25.0	Opportunistic	Damaged fins, mortality Jung et al. (2022)	Unknown	
*Corynebacterium tuberculostearicum*	9.1	23.1	16.7	Unknown		Pathobiont	Inflammatory breast disease, pancreatic panniculitis, chronic rhinosinusitis, and surgical site infections Altonsy et al. (2020)
*Acinetobacter johnsonii*	0.0	38.5	8.3	Opportunistic	Eye haemorrhages, scale loss Kozińska et al. (2014)	Unknown	
*Myroides odoratus*	9.1	0.0	33.3	Opportunistic	Severe skin and soft tissue infections Jacobs and Chenia (2009)	Opportunistic	Necrotizing fasciitis Crum‐Cianflone, Matson, and Ballon‐Landa (2014)
*Cutibacterium acnes*	9.1	0.0	25.0	Unknown		Pathobiont	Acne vulgaris Castillo, Nanda, and Keri (2019)
*Staphylococcus aureus*	9.1	7.7	16.7	Yes	Congestion and haemorrhage of gills, internal organs Zhang et al. (2019)	Opportunistic	Abscesses Pollitt et al. (2018)
*Leucothrix mucor*	0.0	30.8	0.0	Yes	Common fouling agent in aquaculture, causes larval mortality in lobsters Shields (2011)	Unknown	
*Carnobacterium inhibens*	18.2	7.7	0.0	Unknown		Rarely	Bacteremia Lo and Sheth (2021)
*Arcobacter cryaerophilus*	0.0	15.4	0.0	Unknown		Yes	Enteritis, colitis, septicemia van den Abeele et al. (2016)

*Note:* Marine pathogenicity is scaled from ‘No’, through ‘Unknown’, ‘Rarely’, ‘Opportunistic’, ‘Pathobiont’, ‘PP’ (meaning Potentially Pathogenic) to ‘Yes’, as defined in Itay et al. (2022).

Abbreviations: GLF, Greater lizardfish; JTB, Japanese threadfin bream; SHS, Shrimp scad.

**FIGURE 7:**
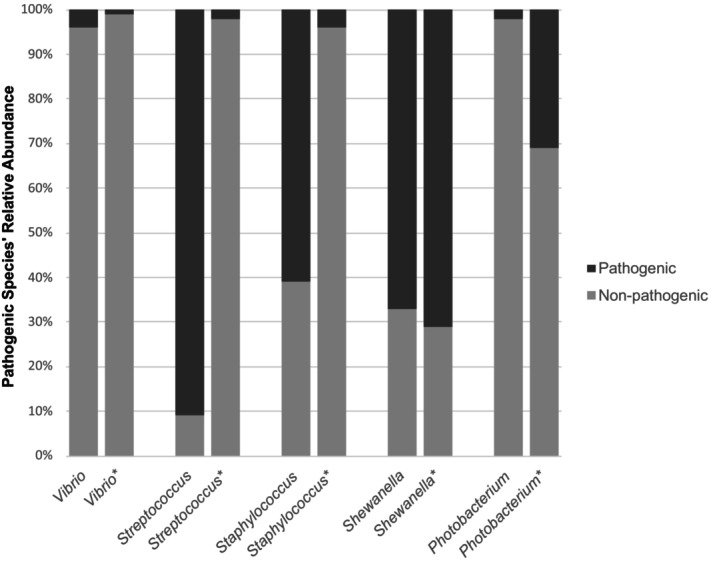
A comparison of five genera of interest that were mentioned in Itay et al. (2022) and their relative abundance of pathogenic species. The left column of each genera represents findings from the current study, while the right column (genus name with an ‘*’) represents that of the previous study referred to above.

A comparison between the results of the current study with that of Itay et al. (2022) regarding Pathogenic/Non‐pathogenic ratios of several genera of interest, is depicted in Figure 7. These genera include: *Photobacterium*, *Shewanella*, *Staphylococcus*, *Streptococcus, and Vibrio*. Phylogenetic trees of these genera (from the current study) are available in the Supporting Information (Figures S1–S5).

## 4 Discussion

The Japanese threadfin bream (JTB; *Nemipterus japonicus*) and the Greater lizardfish (GLF; *Saurida tumbil*) are demersal fish species found throughout the Indian Ocean. Both species feed mainly on crustaceans, squid and smaller fish. The key difference in their habitat preferences is that GLF inhabits depths of 20–60 m, while JTB resides at depths of up to 200 m (Doustdar, Hashemi, and Rahmati 2022; Saraswati and Perdhana 2020; SriHari et al. 2021; Tonnie et al. 2018). This difference in habitat may help explain the variation in gill microbiome diversity and the ratio of similarities to differences between the species.

The shrimp scad (SHS; *Alepes djebaba*) is distributed across the Indian Ocean—from the coasts of East Africa, through South Asia, and as far as Northern Australia. Some populations are even seen in the Pacific Ocean around Hawaii (Quayed et al. 2022). Since the opening of the Suez Canal, this species has been amongst the Lessepsian migrates that have successfully established themselves along the southeastern Mediterranean Sea. SHS is a pelagic species typically found at depths of 60–80 m, though it is also amphidromous, feeding on Zooplankton, fish eggs, and juvenile small fish (Bandkar et al. 2022). Its ability to migrate across diverse geographic regions with varying salinity levels, and of its broad diet, may explain both the richness of its microbiome and the large number of potential pathogens present in its gills.

There are three main surfaces of interaction between the fish and its environment: The gut, the skin, and the gills. Each of these organs harbours its own microbial community and plays a different role in host immunity. The gills, in constant contact with the surrounding water and its associated microbes, serve both as a barrier and a gateway into the fish's body, and are a critical site of mucosal immunity (Salinas 2015). To a certain extent, the gill microbiome may reflect the microbial composition of the water in which the fish resides, including potential pathogens (Kuang et al. 2020).

From an evolutionary perspective, a richer, more diverse gut microbiome improves the host's metabolic performance, enabling it to digest food more efficiently, and possibly creating novel metabolic capabilities (Gomez, Sunyer, and Salinas 2013; Koppang, Kvellestad, and Fischer 2015; Maynard et al. 2012). This, in turn, allows the fish to adopt a more diverse diet, providing the advantage of better coping with rapid environmental changes, such as those experienced by marine species over the past few decades. As a result, species like the shrimp scad are expected to be less vulnerable than those with more restricting feeding habits and habitats. Following the evolutionary path of microbiome diversity and its benefits, one may ask whether the microbiome can also serve as an indicator for assessing vulnerabilities of various fish species to habitat changes driven from the climate crisis. In an intertwined ecological environment such as the sea, the combined stress by multiple factors is both visible and measurable. This makes the work of marine scientists more crucial than ever, providing decision makers with a growing body of hard data for policy‐making.

Fish gill microbiome analyses as a tool for assessing pathogen prevalence has become more widespread in recent years, with its effectiveness gradually becoming more evident (Brown, Wiens, and Salinas 2019; Hess et al. 2015; Minich, Petrus et al. 2020; Minich, Poore et al. 2020; Rosado et al. 2019). In this study, we examined the gills microbiomes of three common wild fish species from the South China Sea. The results demonstrate that potential pathogens are present in the gill microbiota of these fish, with significant diversity in the bacterial species observed. However, the presence does of these pathogens does not necessarily indicate a high potential of disease development. These seemingly contradicting notions can coexist because potential pathogens express virulence only under specific, sometimes extreme, conditions. For example, the presence of *Photobacterium damselae* in large numbers in GLF does not mean these fish were diseased. *P. damselae* may be a pathobiont of the GLF, often beneficial by protecting the fish from other pathogens—while retaining the ability to express virulence under stress or threat (Itay et al. 2022). Conversely, *Shewanella baltica* and *Shewanella frigidimarina* are not considered pathogenic to marine organisms, but are leading causes of food spoilage (Wright et al. 2019; Zhu et al. 2015). Despite being freshly bought from the market just hours after being caught, most of the sampled fish harboured these bacteria in their gills (*S. frigidimarina*: 66.7% in GLF, 100% in both JTB and SHS samples; *S. baltica*: 58.3%, 81.8%, and 100% in GLF, JTB, and SHS, respectively). These findings warrant further research to assess potential risks to public health.

This study was designed to be comparable to that of Itay et al. (2022), using the same methods but on different fish species and in a different geographical location. While repeating the process strengthens the claim regarding the method's feasibility as a cost‐effective way to gather information and make predictions, the comparison has its limitations: (i) the fish were not exactly of the same species; (ii) samples were not collected with a seasonality consideration; and (iii) the previous study used fish captured during a research vessel expedition, while those in the current study came from the fish market (i.e., handling of the fish was less controlled, both in transportation practices and the possibility of sorting out unwanted samples). Nonetheless, some meaningful trends emerge from both studies:

- The Greater lizardfish (GLF; *Saurida tumbil*) is closely related to the Lessepsian lizardfish (LLF; *Saurida lessepsianus*). Analyses of both species revealed a Simpson index indicating high variance between individual samples and medium‐low bacterial diversity (0.75 in LLF; 0.45 in GLF). Both species hosted a significant percentage of the total potentially pathogenic species identified in their respective studies—27/41 (66%) for LLF, 16/20 (80%) for GLF—with *Shewanella baltica* and *Photobacterium damselae* prevalent in many samples (45.8% and 37.5%, in LLF; 58.3% and 66.7% in GLF);
- Both Atlantic chub mackerel (*Scomber colias*; ACM) and Shrimp scad (*Alepes djebaba*; SHS) are migratory species that inhabit vast geographic regions and encounter diverse habitat conditions. These species exhibited the largest variety of potential pathogens (35/41, 85%, in ACM; 18/20, 90% in SHS) and the highest Simpson index averages (0.9 in ACM; 0.91 in SHS), with minimal variance amongst samples.

Another important aspect is the comparison of potential pathogens identified in both studies. In the previous study, 41 potential pathogens were found in fish gills, whereas the current study identified only 20. Besides *S. baltica* and *P. damselae*, only *Shewanella putrefaciens* and *Cutibacterium acnes* were identified in both studies. Several genera, including *Acinetobacter*, *Aeromonas*, *Corynebacterium*, *Staphylococcus*, *Streptococcus*, *Tenacibaculum*, and *Vibrio* were represented by potentially pathogenic species in both studies. The data, visualised in Figure 6, show that *Shewanella* species responsible for food spoilage comprised about two‐thirds of the total *Shewanella* reads in both studies. Pathogenic *Vibrio* species, in both cases, were present in low single‐digit percentages. However, *Staphylococcus* and *Streptococcus* exhibited pathogenic read ratios of 0.61 and 0.91 (respectively), though their percentage of overall abundance was negligible. In contrast, *Photobacterium* played a more significant role in the gill microbiome in both studies, but in this study *Photobacterium damselae* accounted for only 2% of the total *Photobacterium* reads, compared to 31% in the previous study.

To understand the cause of these differences—whether due to geographic spread, host biology, environmental factors, or microbiome composition—it will be necessary to repeat the study in both locations, sampling similar fish, over multiple seasons and years.

## 5 Conclusions

Fish gills harbour species microbiomes, exhibiting solid correlations between certain taxonomic groups. Some overlap exists between the three species sampled, perhaps expressing some form of core microbiome. This methodology, using NGS to collect data from fish gills regarding their microbiota, can be made more valuable if it is made as part of a continuous monitoring program, being either repeated for several consecutive years, being compared with similar spatiotemporal studies or expanded to more fish species. At the same time, it should also be placed in a broader context of the whole studied ecosystem and connected to broader data of marine organisms of higher and lower trophic levels and the physio—geo—bio—chemical data of the ambient water. By doing so, scientists would be able to assert possible connections between changes in abiotic factors such as water temperature, acidity, and oxygen levels, the presence of certain pollutants, etc., with shifts in fish gill microbiota, leading to better predictions on the level of risk of disease outbreaks occurring, thus leading to better management of the marine environment in whole and fish as a vital food resource in specific.

## Author Contributions

**Shlomi Zrihan:** conceptualization, investigation, formal analysis, writing – original draft, resources. **Peleg Itay:** data curation, methodology, formal analysis, visualization, writing – review and editing, writing – original draft. **Yael Kroin:** investigation, formal analysis. **Nadav Davidovich:** conceptualization, investigation, formal analysis. **Natascha Wosnick:** conceptualization, writing – review and editing, supervision. **Dan Tchernov:** conceptualization, funding acquisition, supervision, resources, writing – review and editing. **Xiu Pei Koh:** investigation, writing – review and editing. **Stanley C. K. Lau:** conceptualization, investigation, funding acquisition, writing – review and editing, supervision, resources. **Danny Morick:** conceptualization, investigation, funding acquisition, writing – original draft, writing – review and editing, methodology, formal analysis, supervision, resources, validation.

## Ethics Statement

This work has received approval for research ethics from The Ministry of Environmental Protection, Israel, and a proof/certificate of approval is available upon request.

## Conflicts of Interest

The authors declare no conflicts of interest.

## Supporting information


**Figure S1** A phylogenetic tree for *Photobacterium*‐related ASVs. A cutoff of 0.7 (70% bootstrap support) was made for nodes, thus any lower value is not presented. Triangular shaped tips represent sequences found to be practically identical. Reference sequences include their GenBank accession numbers. Smaller ASV numbers indicate they were more common (in terms of total reads) than ASVs with large numbers. The scale bar represents 0.1 nucleotide substitution per site.
**Figure S2** A phylogenetic tree for *Shewanella*‐related ASVs. A cutoff of 0.7 (70% bootstrap support) was made for nodes, thus any lower value is not presented. Triangular shaped tips represent sequences found to be practically identical. Reference sequences include their GenBank accession numbers. Smaller ASV numbers indicate they were more common (in terms of total reads) than ASVs with large numbers. The scale bar represents 0.1 nucleotide substitution per site.
**Figure S3** A phylogenetic tree for *Staphylococcus*‐related ASVs. A cutoff of 0.7 (70% bootstrap support) was made for nodes, thus any lower value is not presented. Triangular shaped tips represent sequences found to be practically identical. Reference sequences include their GenBank accession numbers. Smaller ASV numbers indicate they were more common (in terms of total reads) than ASVs with large numbers. The scale bar represents 0.1 nucleotide substitution per site.
**Figure S4** A phylogenetic tree for *Streptococcus*‐related ASVs. A cutoff of 0.7 (70% bootstrap support) was made for nodes, thus any lower value is not presented. Triangular shaped tips represent sequences found to be practically identical. Reference sequences include their GenBank accession numbers. Smaller ASV numbers indicate they were more common (in terms of total reads) than ASVs with large numbers. The scale bar represents 0.1 nucleotide substitution per site.
**Figure S5** A phylogenetic tree for *Vibrio*‐related ASVs. A cutoff of 0.7 (70% bootstrap support) was made for nodes, thus any lower value is not presented. Triangular shaped tips represent sequences found to be practically identical. Reference sequences include their GenBank accession numbers. Smaller ASV numbers indicate they were more common (in terms of total reads) than ASVs with large numbers. The scale bar represents 0.1 nucleotide substitution per site.

Appendix S1 Supplementary methods for: Sequence data processing; Data analysis; Phylogentic trees.

Data S1 Fish dissection data for *Alepes djebaba.*

Data S2 Fish dissection data for *Nemipterus japonicus*.

Data S3 Fish dissection data for *Saurida tumbil*.

## Data Availability

The datasets generated for this study are available upon request to the corresponding author.
