# Supplementary figures and images for: Monitoring Fish Bacterial Pathogens of Wild Fish Species From the South China Sea by Applying Next‐Generation Sequencing on Gill Tissue
Source: J Fish Dis. 2024 Nov 22;48(2):e14050. doi: 10.1111/jfd.14050 (PMC11706320; doi:10.1111/jfd.14050)

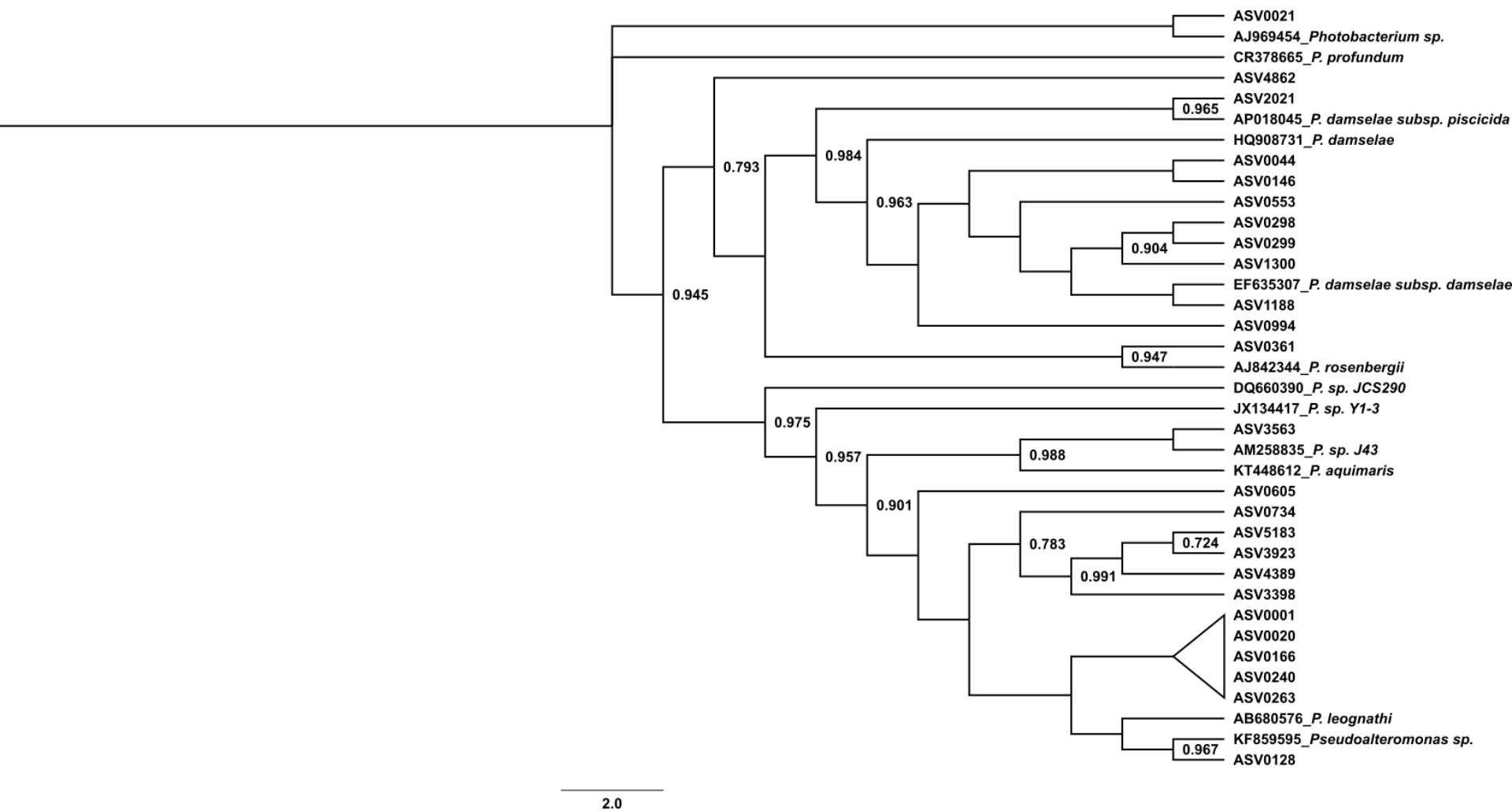

Figure S1

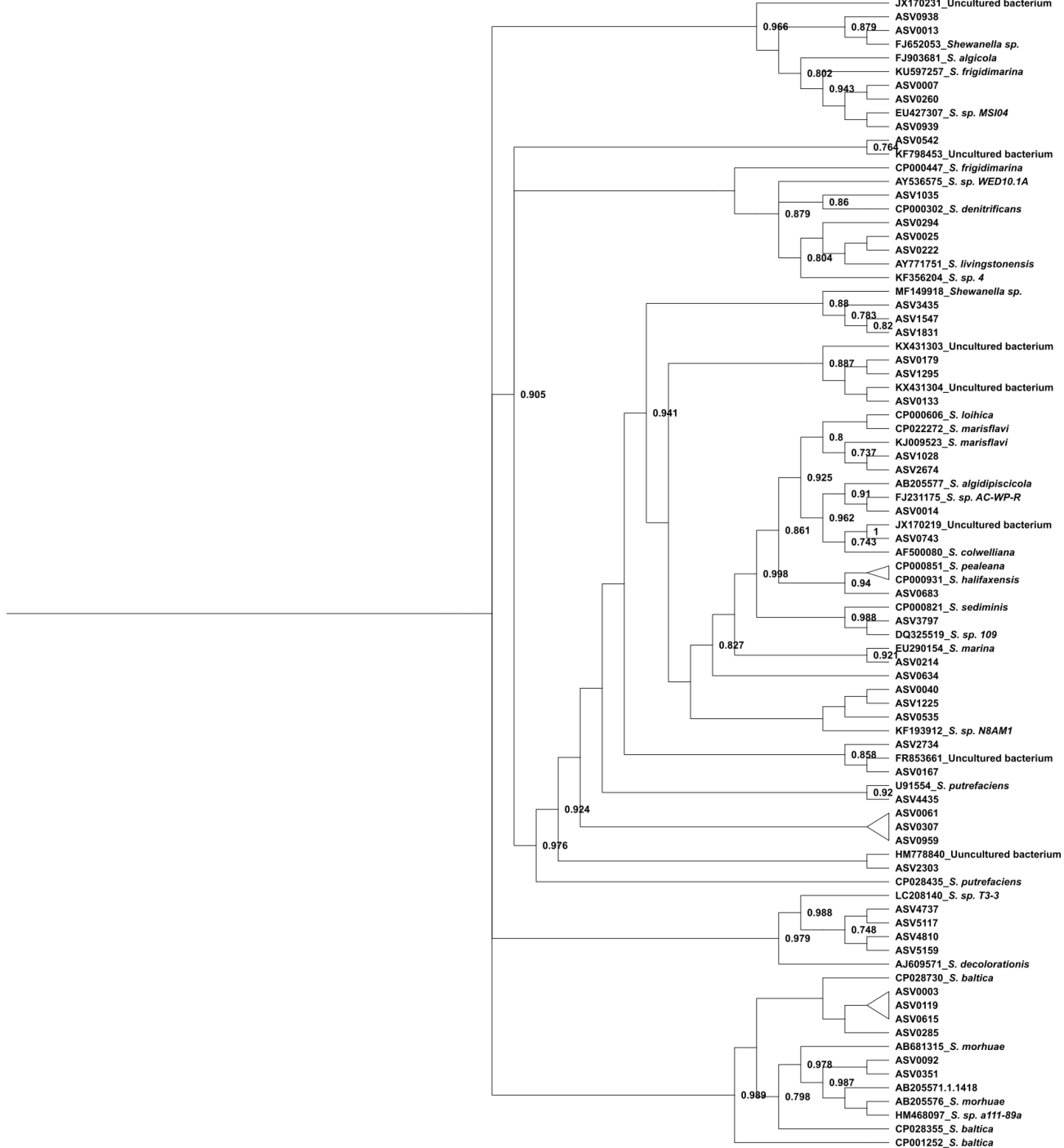

Figure S2

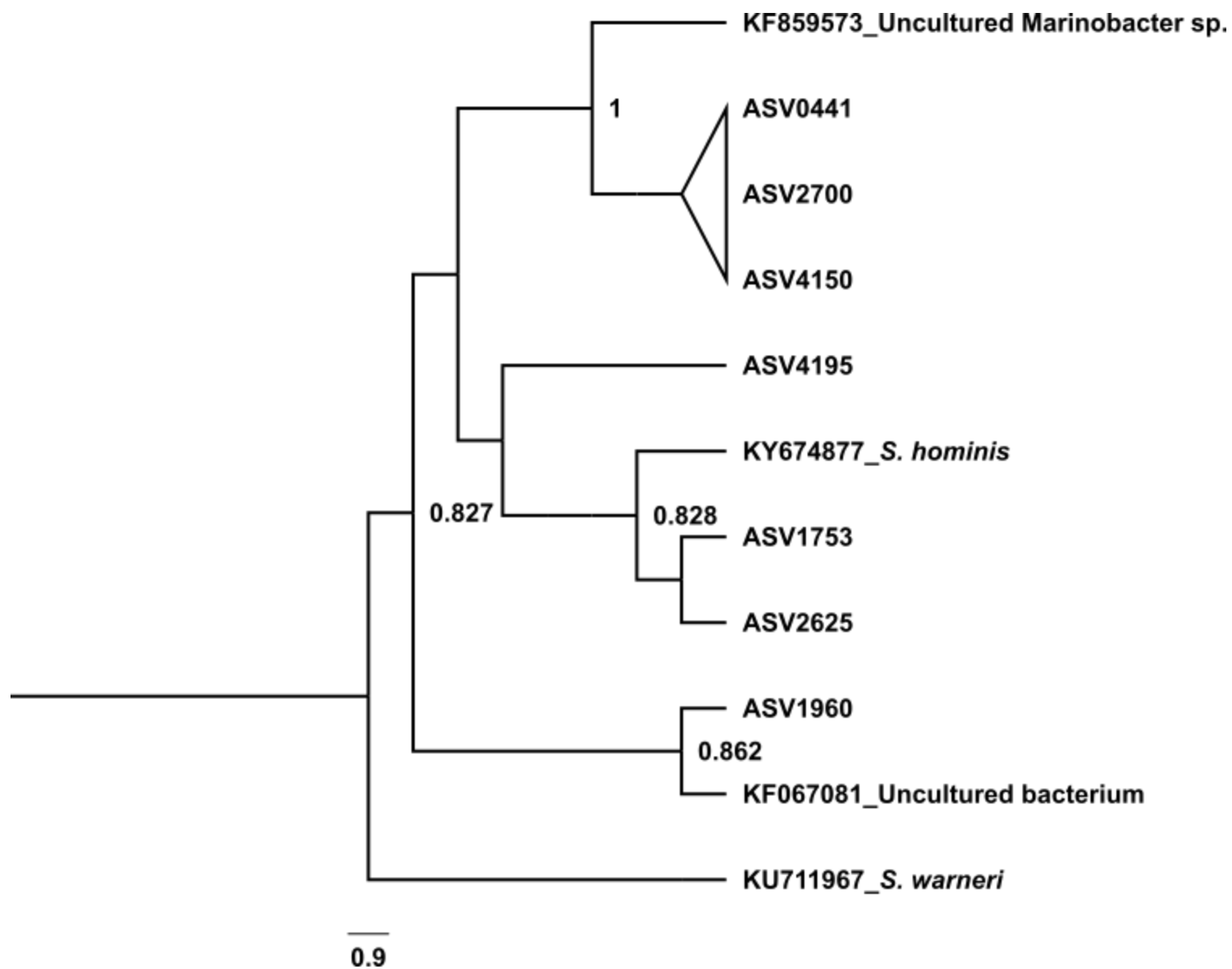

Figure S3

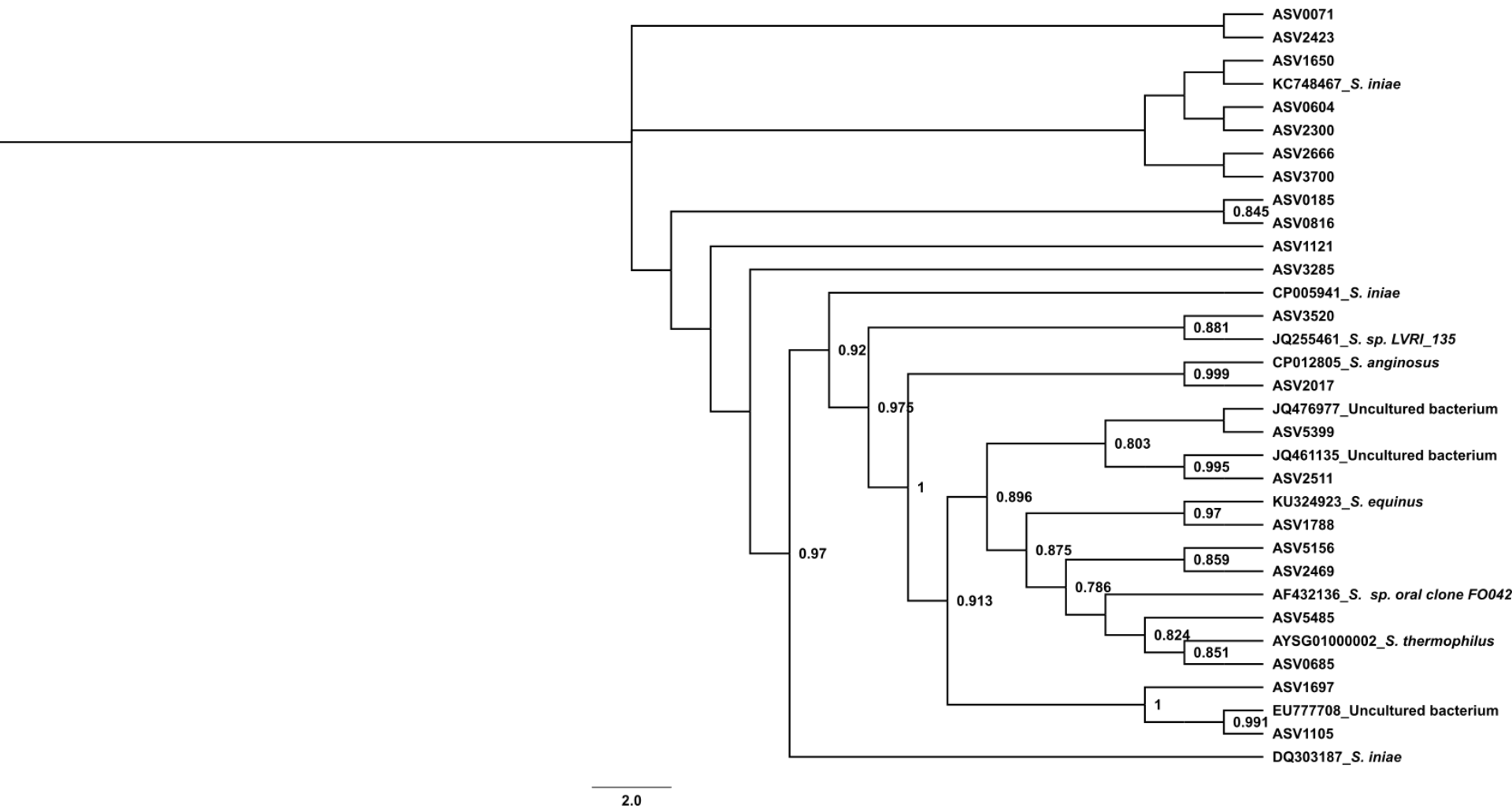

Figure S4

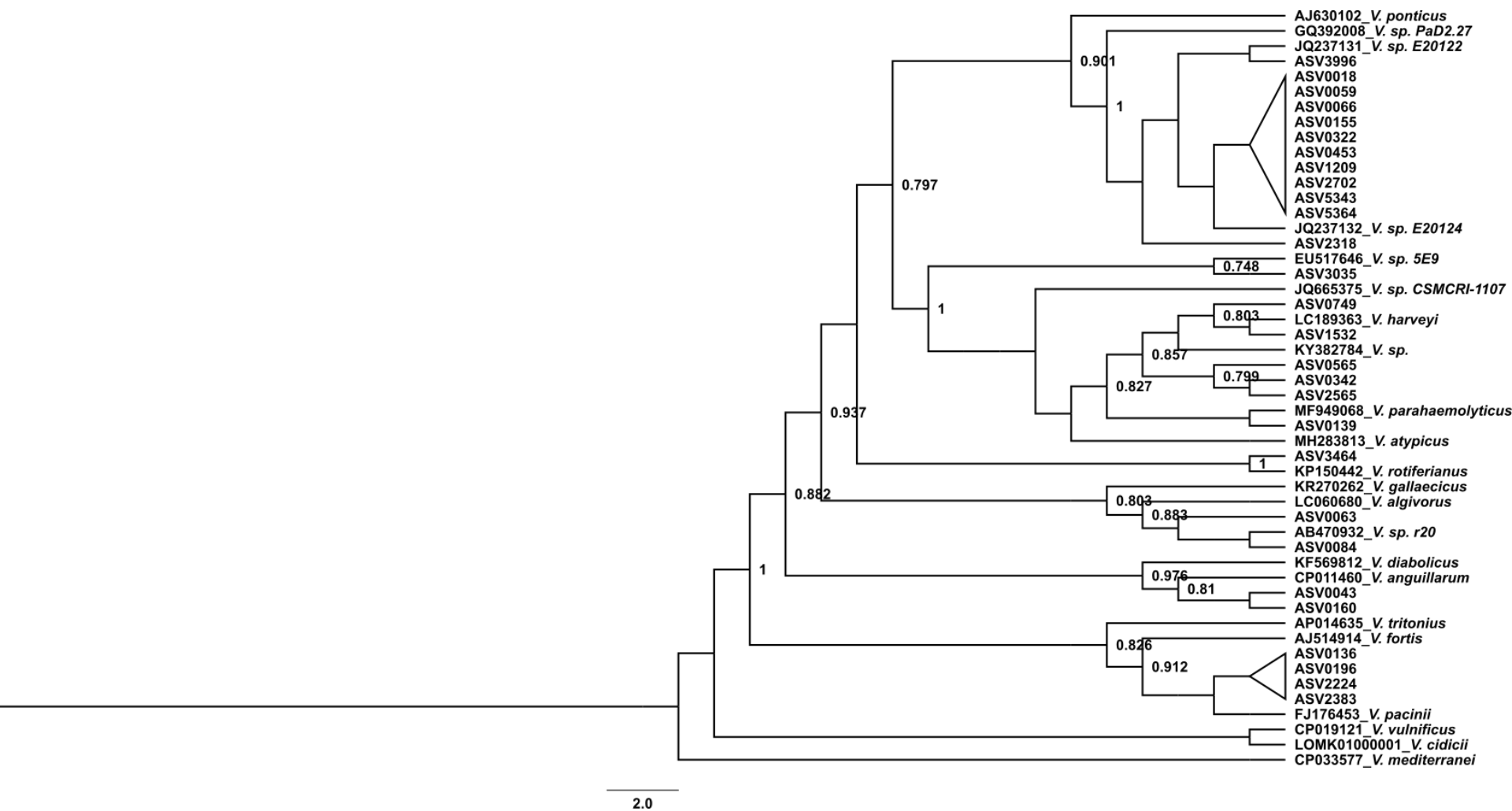

Figure S5

Supplement: Supplementary file 1 — Figure S1 A phylogenetic tree for Photobacterium‐related ASVs. A cutoff of 0.7 (70% bootstrap support) was made for nodes, thus any lower value is not presented. Triangular shaped tips represent sequences found to be practically identical. Reference sequences include their GenBank accession numbers. Smaller ASV numbers indicate they were more common (in terms of total reads) than ASVs with large numbers. The scale bar represents 0.1 nucleotide substitution per site. Figure S2 A phylogenetic tree for Shewanella‐related ASVs. A cutoff of 0.7 (70% bootstrap support) was made for nodes, thus any lower value is not presented. Triangular shaped tips represent sequences found to be practically identical. Reference sequences include their GenBank accession numbers. Smaller ASV numbers indicate they were more common (in terms of total reads) than ASVs with large numbers. The scale bar represents 0.1 nucleotide substitution per site. Figure S3 A phylogenetic tree for Staphylococcus‐related ASVs. A cutoff of 0.7 (70% bootstrap support) was made for nodes, thus any lower value is not presented. Triangular shaped tips represent sequences found to be practically identical. Reference sequences include their GenBank accession numbers. Smaller ASV numbers indicate they were more common (in terms of total reads) than ASVs with large numbers. The scale bar represents 0.1 nucleotide substitution per site. Figure S4 A phylogenetic tree for Streptococcus‐related ASVs. A cutoff of 0.7 (70% bootstrap support) was made for nodes, thus any lower value is not presented. Triangular shaped tips represent sequences found to be practically identical. Reference sequences include their GenBank accession numbers. Smaller ASV numbers indicate they were more common (in terms of total reads) than ASVs with large numbers. The scale bar represents 0.1 nucleotide substitution per site. Figure S5 A phylogenetic tree for Vibrio‐related ASVs. A cutoff of 0.7 (70% bootstrap support) was made for nodes, thus a [file JFD-48-e14050-s004.pdf]
