# Supplementary material for: Monitoring Fish Bacterial Pathogens of Wild Fish Species From the South China Sea by Applying Next‐Generation Sequencing on Gill Tissue
Source: J Fish Dis. 2024 Nov 22;48(2):e14050. doi: 10.1111/jfd.14050 (PMC11706320; doi:10.1111/jfd.14050)
Supplement: Supplementary file 2 — Appendix S1 Supplementary methods for: Sequence data processing; Data analysis; Phylogentic trees. [file JFD-48-e14050-s001.docx]

# Supplementary Information for:

**Monitoring Fish Bacterial Pathogens of Wild Fish Species from The South China Sea by Applying Next-Generation Sequencing on Gill Tissue**

# Supplementary Methods

### Sequence data processing

Sequence data was analyzed using the Dada2 pipeline using R package ‘dada2’ (version 1.14.1). Fastq formatted reads were trimmed and filtered for low quality using the command ‘filterAndTrim’ with parameters maxEE=2, maxN=0, trimleft=20 and the trunclen=150. Error rate estimation was carried out using the ‘learnerror’ command with default parameters, but with the randomize parameter set to TRUE, in order to sample nucleotides and reads for model building randomly across all samples. The dada2 algorithm was implemented for error correction and a count table containing the amplicon sequence variants and counts per sample was produced. Merging of forward and reverse reads was done using the ‘mergePairs’ command with a minimum overlap of 8 bases. Then, suspected chimeras were detected and removed using the command ‘removeBimeraDenovo’, with default parameters. For each amplicon sequence variant (ASV), taxonomy (up to the species level) was inferred by alignment to the Silva non-redundant small subunit ribosomal RNA database (version 138), using commands ‘assignTaxonomy’ and ‘addSpecies’ with default parameters, while setting the minimum bootstrap confidence value to 80%.

### Data analysis

For data analysis and generation of figures, the online tool MicrobiomeAnalyst ([https://www.microbiomeanalyst.ca/MicrobiomeAnalyst/home.xhtml](about:blank)) was used. Through Marker Data Profiling (MDP), three CSV files were uploaded – ASV counts, taxonomy, metadata. Taxonomy labels were assigned using the SILVA taxonomic framework (https://www.arb-silva.de/documentation/silva-taxonomy/). Data filtering settings were defined as follows: (i) minimum counts: 4; (ii) low count filter – prevalence in samples (%): 10; (iii) low variance filter – percentage to remove (%): 5, and filter based on inter-quantile range. This process removed 603 low abundance/variance features and kept 340 others. For Alpha-diversity graphs data normalization settings were: (i) data rarefied to the minimum library size; (ii) no data scaling; and (iii) no data transformation. Alpha-diversity profiling settings were: (i) data input – filtered; (ii) experimental factor – 'species'; (iii) taxonomic level – 'feature-level'; (ⅳ) diversity measure – observed, Shannon and Simpson; and (v) the statistical method – Mann-Whitney/Kruskal-Wallis. For all other graphs and analyses, the data normalization settings required not rarefying data, but transforming the data using relative log expression (RLE). For taxa abundance stacked-bar graphs, graph type was set to percentage-abundance, merging small taxa with counts < 4,000 and grouping by fish species. For beta-diversity graphs, method: NMDS ordination, distance method calculation: Jaccard index. Statistical method: Permutational MANOVA (PERMANOVA), other options kept as default. For the correlation analysis: algorithm – Spearman's rank correlation, *P*-value threshold – 0.05, correlation threshold – 0.5. All other values used default settings.

### Phylogenetic trees

Sequences identified as belonging to the several genera chosen for deeper enquiry were copied from the main data file converted to FASTA format, and then uploaded to Silva ([https://www.arb-silva.de/aligner/](about:blank)) for preparing phylogenetic files. The ACT (Alignment, Classification and Tree Service) tool was used (SINA v1.2.11), with the following parameters: (i) gene: ssu; (ii) unaligned remaining bases – attached to the last aligned base; (iii) search and classify (checked) with minimum identity with query sequence set to 0.98, and number of neighbors per query sequence at 2; (ⅳ) compute tree (checked) with its workflow set to 'denovo including neighbors', 'FastTree' as the program to use, and 'gamma' as the rate model for likelihoods; (v) the output format: FASTA and file zip-compressed; and (vi) taxonomies selected for classification: SILVA and RDP (only). All other parameters were kept as default. Output TREE format files were extracted for visualization with the FigTree v1.4.3 software ([http://tree.bio.ed.ac.uk/software/figtree/](about:blank)).
